# Surgical Patients Admitted to Intensive Care: Do Elective and Urgent Surgical Patients Have Similar Outcomes?

**DOI:** 10.7759/cureus.103644

**Published:** 2026-02-15

**Authors:** Ana M Oliveira, Inês Simões, André C Oliveira, Ana Martinho, Rui Morais, João Gonçalves-Pereira

**Affiliations:** 1 Intensive Care Unit, Hospital Vila Franca de Xira, Vila Franca de Xira, PRT; 2 Medicine, Universidade de Lisboa, Lisboa, PRT; 3 Intensive Care Unit, Centro Hospitalar e Universitário de Coimbra, Coimbra, PRT; 4 Intensive Care Unit, Hospital São Francisco Xavier, Lisboa, PRT; 5 Infection and Sepsis, Grupo de Investigação e Desenvolvimento em Sépsis, Lisboa, PRT

**Keywords:** elective surgery, intensive care unit outcomes, long-term mortality, sepsis, standardized mortality ratio, urgent surgery

## Abstract

Background: The landscape of critical care is evolving. Advances in treatment and supportive care, along with the evolving technology supporting surgical interventions, such as robotics, have made it possible to achieve outstanding results. Contemporary evaluation of prognosis in critically ill surgical patients is paramount to support individualized care and informed decision-making. This study aimed to assess and compare short-term (first 30 days) and long-term (up to two years) outcomes of elective versus urgent surgical patients admitted to the intensive care unit (ICU) and to characterize their clinical trajectory patterns beyond the traditional grouping of “surgical patients.”

Methods: We performed a post hoc analysis of the Critically Ill patients’ mortality by age: Long-Term follow-up (CIMbA-LT) study, a retrospective, multicenter, observational study conducted on Portuguese multipurpose ICUs over four years. Surgical patients were segregated for further analysis. We compared differences between patients admitted after elective or urgent surgery.

Results: We included 13,748 surgical adult patients admitted to an ICU during the study period. Roughly one-third underwent elective procedures and two-thirds urgent surgery. Patients submitted to an urgent procedure presented higher severity and in-hospital mortality (24.6% versus 8.2%, p<0.001), while among those discharged alive, scheduled surgery patients had higher long-term mortality (28.9% versus 25.0%, odds ratio (OR): 1.21, 95% confidence interval (CI): 1.11-1.32). This difference in the long-term risk was statistically significant in the younger population. All surgical critically ill patients had a markedly increased risk of one-year mortality, compared to the general population, even after hospital discharge (urgent surgery: OR: 10.8, 95% CI: 9.0-12.9; elective surgery: OR: 14.6, 95% CI: 11.3-18.8).

Conclusions: Elective and urgent surgical patients admitted to the ICU demonstrate distinct clinical trajectories and require tailored follow-up strategies. Urgent surgery patients have higher short-term mortality, while elective surgical patients showed higher long-term mortality risk, particularly noticeable in younger cohorts. All surgical patients admitted to the ICU had a very high first-year risk of mortality.

## Introduction

The critical care population is changing. Improvements in treatment sophistication and better supportive intensive care have facilitated the achievement of treatment goals once considered unthinkable. Moreover, the advent of artificial intelligence seems to be leading to additional gains in the quality of care [1,2]. Consequently, fewer limitations are imposed on patients who need invasive treatments, even in those who are old and frail or have significant comorbidities. Complex surgical procedures are now possible for high-risk patients, supported by new technologies (e.g., robotics) and increasingly efficient organ support.

Critically ill patients who require close observation, monitoring, and treatment are frequently admitted to the intensive care unit (ICU). A substantial percentage of ICU admissions is attributable to postoperative status. Admission to an ICU can improve the prognosis of surgical patients and lower costs by preventing or treating life-threatening complications that may occur after surgery [3,4].

However, there is scarce information on the short- and long-term prognosis of these patients after ICU admission according to the type of surgical procedure, particularly the differences between elective, scheduled, and urgent, unscheduled surgery [5]. This information is of the utmost importance to support informed decisions, enhance patient and family autonomy, and plan individualized follow-up.

Elective surgery is a procedure performed on patients admitted for scheduled intervention. Hospital mortality is usually low (<1%), but heterogeneity may be observed [4]. Outcomes are usually measured as early as hospital discharge, and late mortality is often unknown.

On the contrary, general surgical procedures performed urgently may reach a 12.3% morbidity rate and 2.3% mortality. Emergency procedures carry even higher risk (morbidity: 13.8%, mortality: 3.7%) compared with elective surgery (morbidity: 6.7%, mortality: 0.4%) [6]. However, this must be weighed according to the host characteristics, especially age and comorbidities. Emergency surgery is associated with increased complication rates, including medical errors, organ failure due to hypoperfusion and over-inflammatory response, and death, as compared with elective procedures [7].

Postoperative admission to the ICU may facilitate prevention, early detection, and prompt treatment of potentially fatal complications arising immediately after surgery [8,9]. Nevertheless, there is insufficient data to support a universal benefit from ICU admission [10], and its impact has recently been challenged [11]. Besides, a long ICU stay may lead to worse outcomes, both after elective and urgent surgery, particularly in older patients [12]. Differences in surgical populations may foster the need for a personalized approach, as patients undergoing urgent procedures may benefit more from intensive monitoring but may also be at increased risk of post-intensive care syndrome [13]. Conversely, elective surgical patients’ care may focus more on the long-term effects of the surgical disease, suggesting that grouping all individuals simply as “surgical patients” may not be appropriate.

This study aimed to assess and compare short- and long-term outcomes of elective versus urgent surgical patients admitted to the ICU, in order to characterize their clinical trajectories according to age and disease severity at ICU admission.

## Materials and methods

Study protocol

This was a post hoc analysis of the Critically Ill patients’ mortality by age: Long-Term follow-up (CIMbA-LT) study, a retrospective, multicenter, observational study addressing short- and long-term prognosis of ICU patients conducted on Portuguese multipurpose ICUs. The study protocol has been published elsewhere [14].

Briefly, all adult patients (N=37,118) admitted to one of the 16 participating ICUs between January 2015 and June 2019, for more than 24 hours, were included. Data collected at ICU admission included age, gender, main cause of admission (medical or surgical, and elective or urgent), the Simplified Acute Physiology Score (SAPS) II severity score [15], and the presence of sepsis. Sepsis was defined clinically, based predominantly on Sepsis-2 criteria, and assessed locally by the physician in charge at each participating center.

All patients were followed until hospital discharge (either dead or alive) and throughout the first two years post-admission. The Unidade Local de Saúde Estuário do Tejo Ethics Committee approved the study protocol (October 1, 2020). All participating centers’ ethics committees approved the study and the center’s participation. Informed consent was waived due to the observational, retrospective nature of the study and the potential risk of bias if an excess of deceased patients was excluded. Only aggregated data were used.

All data were collected locally in a special file created for the study. The same were merged into a central file after anonymization. The global file was only accessible to the principal investigators. Data for this study were selected from the main file. Patients with significant missing data or implausible outliers were excluded from further analysis.

Collected data

The included patients were classified as medical or surgical according to the SAPS II criteria. Surgical patients were further categorized as urgent or elective. Urgent surgery was defined as ICU admission following a surgical procedure performed within the previous seven days that had not been scheduled more than 24 hours before admission. Elective surgery was defined as a procedure scheduled more than 24 hours before ICU admission. Patients with no surgical procedure within one week of admission were classified as medical.

For each group, the SAPS II severity score, age, gender, and the presence of sepsis were screened. Outcomes of interest were the ICU and the in-hospital length of stay (LOS) and mortality. Outcomes were assessed at the ICU discharge, hospital discharge, and during follow-up, after completing the first year and the second year after ICU admission. All patients were classified as dead or alive. Differences between Portuguese regions (north, center, islands, or south) were assessed.

Mortality

Mortality was evaluated according to the type of admission, age group, and the presence of sepsis. The odds ratio (OR) was computed with 95% confidence interval (CI) for all groups of interest.

The population was further divided into four categories based on their age at ICU admission: adults (18-50 years), seniors (51-65 years), old adults (66-80 years), and very old adults (81 years and older). Kaplan-Meier survival curves were plotted for the first 30 days after ICU admission and, for patients who survived the first month after ICU admission, until two years of follow-up. Patients were divided according to age group and the type of surgery (elective versus urgent). Differences between groups were computed using the log-rank test.

Standardized mortality

The standardized mortality ratio (SMR) was calculated for every group of interest, defined as the ratio between observed mortality and the SAPS II predicted hospital mortality. The 95% CI were computed. Although the SMR was validated only for hospital mortality, we calculated the same ratio for all the time points, including two years of follow-up.

The Portuguese national statistics (publicly available from https://www.ine.pt) were assessed and used to calculate the one-year all-cause mortality of a standard population with the same gender and age. That served as a comparator for elective and urgent surgical patients discharged from the hospital.

This study followed the Strengthening the Reporting of Observational Studies in Epidemiology (STROBE) checklist for observational studies (https://www.strobe-statement.org/check lists/).

Statistical analysis

Descriptive statistics were computed. Data were summarized as mean ± standard deviation (SD) or median (25%-75% interquartile range (IQR)), according to data distribution. Categorical variables were described as numbers (%). The chi-square test was used to compare categorical variables, while continuous variables were evaluated with Student’s t-test or Kruskal-Wallis test, according to data distribution. ORs with 95% CI were computed. A Cox proportional hazard model was used to assess the impact of sepsis on admission on long-term mortality risk.

We calculate our statistical power to assess differences in short- and long-term mortality according to age group. For the two younger groups, assuming a baseline mortality of 5%, we had an >85% power to detect a mortality difference of 3%, both for short- and long-term mortality. For group 3, assuming a baseline mortality of 10%, we also had an >85% power to detect a 3% difference in mortality. For the older patients, again assuming a 10% baseline mortality, we had an >80% power to detect a 5% difference in mortality.

Statistical analysis was performed using IBM SPSS Statistics v.29.0 (IBM, Somers, NY). All statistics were two-tailed, and the significance level was set at p<0.05.

## Results

Demographics and baseline characteristics

Data were collected from 16 different centers in Portugal, which included about 65% of all available beds from Portuguese ICUs, at the time of the study.

The sample included 37,118 patients, of whom 23,370 (63%) were classified as medical patients, 9,359 (25.2%) were surgical urgent patients, and 4,389 (11.8%) were surgical elective patients. Significant heterogeneity was noted nationwide, with a rate of medical patients between 43.5% and 85.2% (mean: 63.3%) and a rate of elective surgery patients admitted to the ICU between 0.1% and 25.2% (mean: 11.8%).

Table 1 shows the patients’ characteristics on ICU admission. Urgent surgical patients had higher severity on admission (measured by the SAPS II severity score) and similar age, and more often presented with sepsis than elective surgical patients. Besides, both ICU LOS and total hospital LOS (from ICU admission until hospital discharge) were significantly longer in urgent surgical patients.

**Table 1:** Patients’ characteristics according to the type of surgery Values are presented as mean ± standard deviation, median (interquartile range), or number (percentage), as appropriate. SAPS: Simplified Acute Physiology Score, LOS: length of stay, ICU: intensive care unit, H: hospital, SD: standard deviation, IQR: interquartile range *Pearson chi-square test (X2) **Student’s t-test #Mann-Whitney U test ^a^Cumulative mortality, during the first two years after intensive care unit admission

Variables		Urgent surgical patients (N=9,359)	Elective surgical patients (N=4,389)	Test	Significance level, p
Gender	Male (number (%))	5,668 (60.6%)	2,741 (62.5%)	X^2^=4.49*	0.036*
	Female (number (%))	3,691 (39.4%)	1,648 (37.5%)		
Age, years (mean±SD)		62.7±17.8	63.2±15.0	t=1.485**	0.137**
SAPS II (mean±SD)		44.8±18.9	28.1±15	t=-55.5**	<0.001**
Sepsis (number (%))		3,026 (32.3%)	360 (8.2%)	X^2^=952.84*	<0.001*
Region, (number (%))	North	3,616 (38.6%)	1,923 (43.8%)	X^2^=129.48*	<0.001*
	South	4,119 (44.0%)	1,933 (44.0%)		
	Center	963 (10.3%)	207 (4.7%)		
	Islands	661 (7.1%)	326 (7.4%)		
LOS (median (IQR))	ICU	5 (8.5)	2 (2.9)	U=11316349^#^	<0.001^#^
	H (total)	16.1 (26.4)	10.1 (14.4)	U=14540474^#^	<0.001^#^
Mortality (number (%))	H	2,306 (24.6%)	361 (8.2%)	X^2^=514.84*	<0.001*
	2-year follow-up^a^	4,072 (43.5%)	1,526 (34.7%)	X^2^=94.56*	<0.001*

Mortality according to the type of admission, age, and presence of sepsis

Higher mortality was noted in patients admitted after an urgent surgical procedure at all time points. The in-hospital mortality (ICU plus ward) was much higher in urgent surgical patients (24.6% versus 8.2%, p<0.001), as predicted by the higher SAPS II severity score. The SMR was 0.66±0.03 for urgent surgery and 0.54±0.06 for elective surgery.

**Figure 1:**
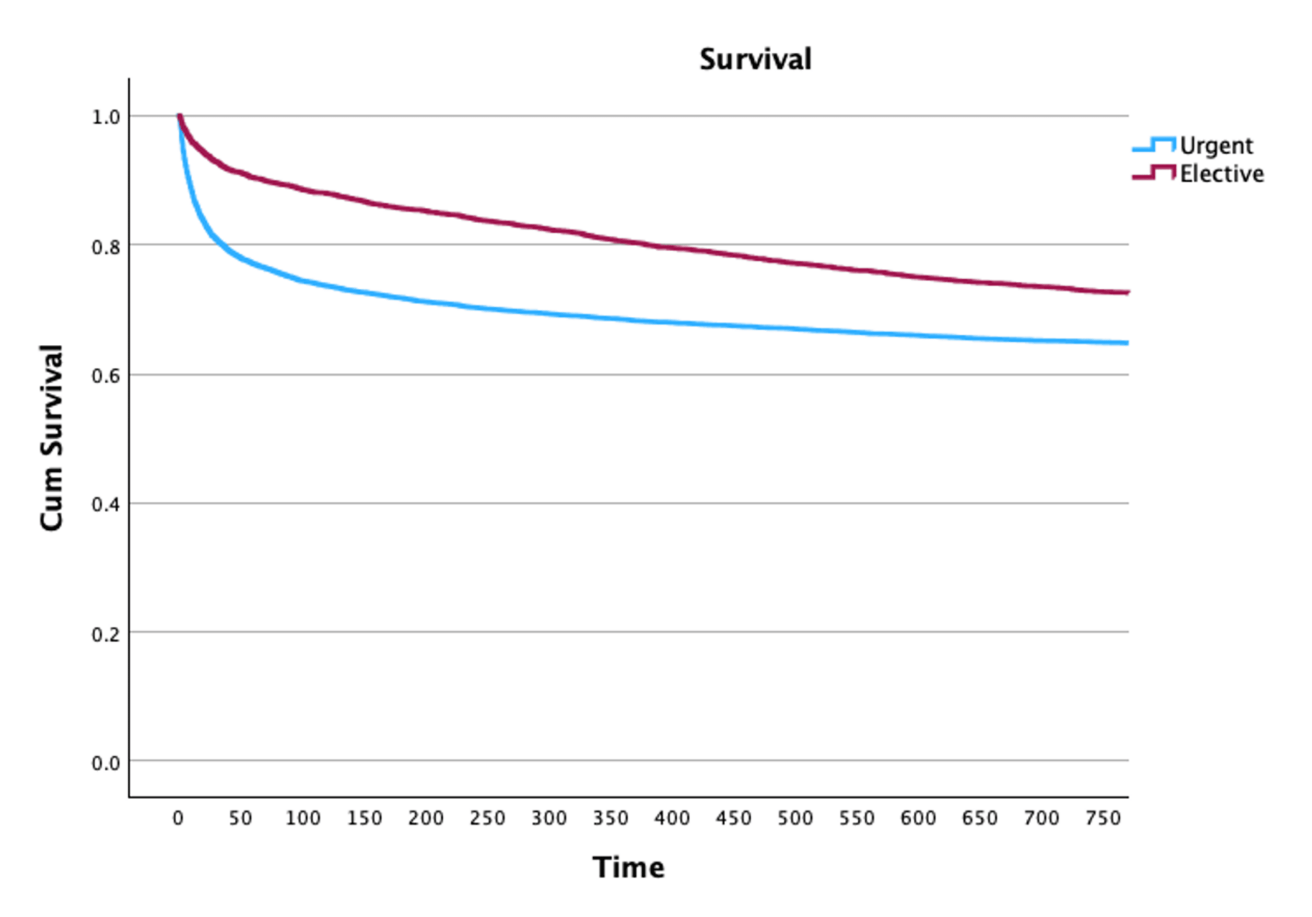
Kaplan-Meier survival curve according to the main reason for ICU admission (ICU admission until two years of follow-up) Log-rank test p<0.001 ICU: intensive care unit

Conversely, when addressing only patients who survived until hospital discharge, the two-year mortality was 20% higher in patients submitted to elective procedures (OR: 1.21, 95% CI: 1.11-1.32) (Table 2 and Figure 1). At that time, the SMR was also higher in elective surgery patients (2.3±0.12 versus 1.17±0.04) (Table 2).

**Table 2:** Mortality of elective and urgent surgical patients Values are presented as number (percentage) or mean ± standard deviation, as appropriate. SMR: standardized mortality ratio, ICU: intensive care unit, OR: odds ratio, CI: confidence interval, SD: standard deviation *Patients discharged alive from the ICU **Patients discharged alive from the hospital

Variables	Elective surgical patients (N=4,389)	Urgent surgical patients (N=9,359)	OR	95% CI
ICU (number (%))	162 (3.7%)	1,526 (16.3%)	0.20	0.17-0.23
Hospital ward (number (%))*	206 (4.7%)	926 (9.9%)	0.45	0.38-0.52
2-year follow-up (number (%))**	1,268 (28.9%)	2,340 (25.0%)	1.21	1.11-1.32
Hospital SMR (mean±SD)	0.54±0.06	0.66±0.03		
2-year SMR (mean ± SD)	2.3±0.12	1.17±0.04		

Standardized mortality

Age was strongly associated with the short- and long-term mortality of patients submitted to either urgent or elective surgery.

**Figure 2:**
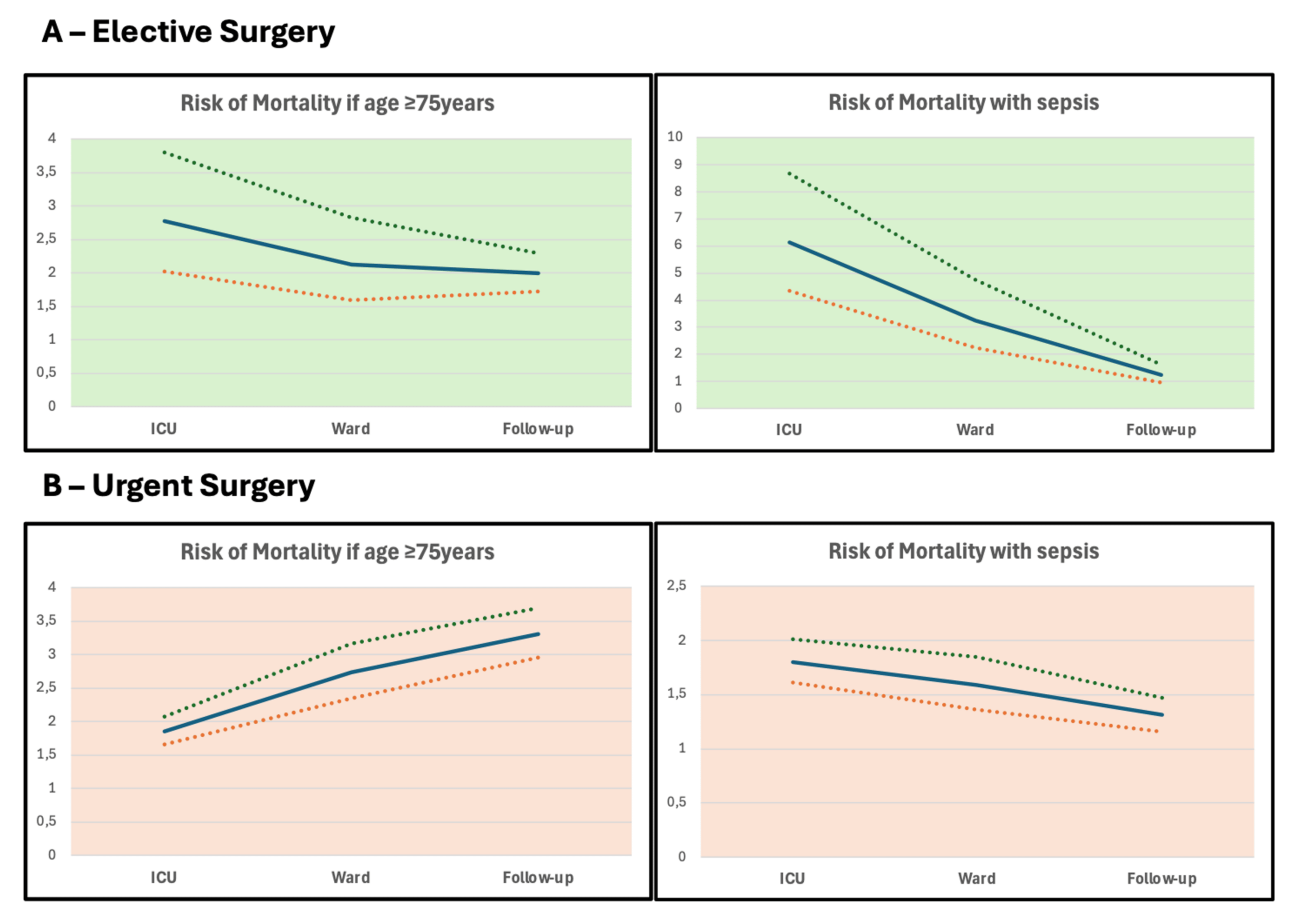
Risk of mortality associated with older age and sepsis for surgical patients, elective (A) and urgent (B), at the ICU, hospital, and after two years (continuous line: odds ratio, dashed lines: 95% confidence interval) ICU: intensive care unit

In Figure 2, we present the timeline of the OR (with 95% CI) for the risk of dying if patients had an older age (>75 years) or sepsis on admission to the ICU, separated according to the type of surgery, elective (Figure 2A) or urgent (Figure 2B). Not surprisingly, the OR for mortality decreased with the time after the septic event, both in elective and urgent surgical patients.

However, although the risk of dying associated with older age also decreases with time after an elective surgery, it increases with time after an urgent surgery (Figure 2).

The presence of sepsis on admission seemed to increase the short- and long-term risk of dying (OR always above 1). However, after adjusting for age and the SAPS II severity score, this risk was no longer significant (Cox proportional hazard: 1.04, 95% CI: 0.98-1.11, p=0.20, for patients with urgent surgery; Cox proportional hazard: 1.16, 95% CI: 0.99-1.04, p=0.07, after elective surgery).

**Figure 3:**
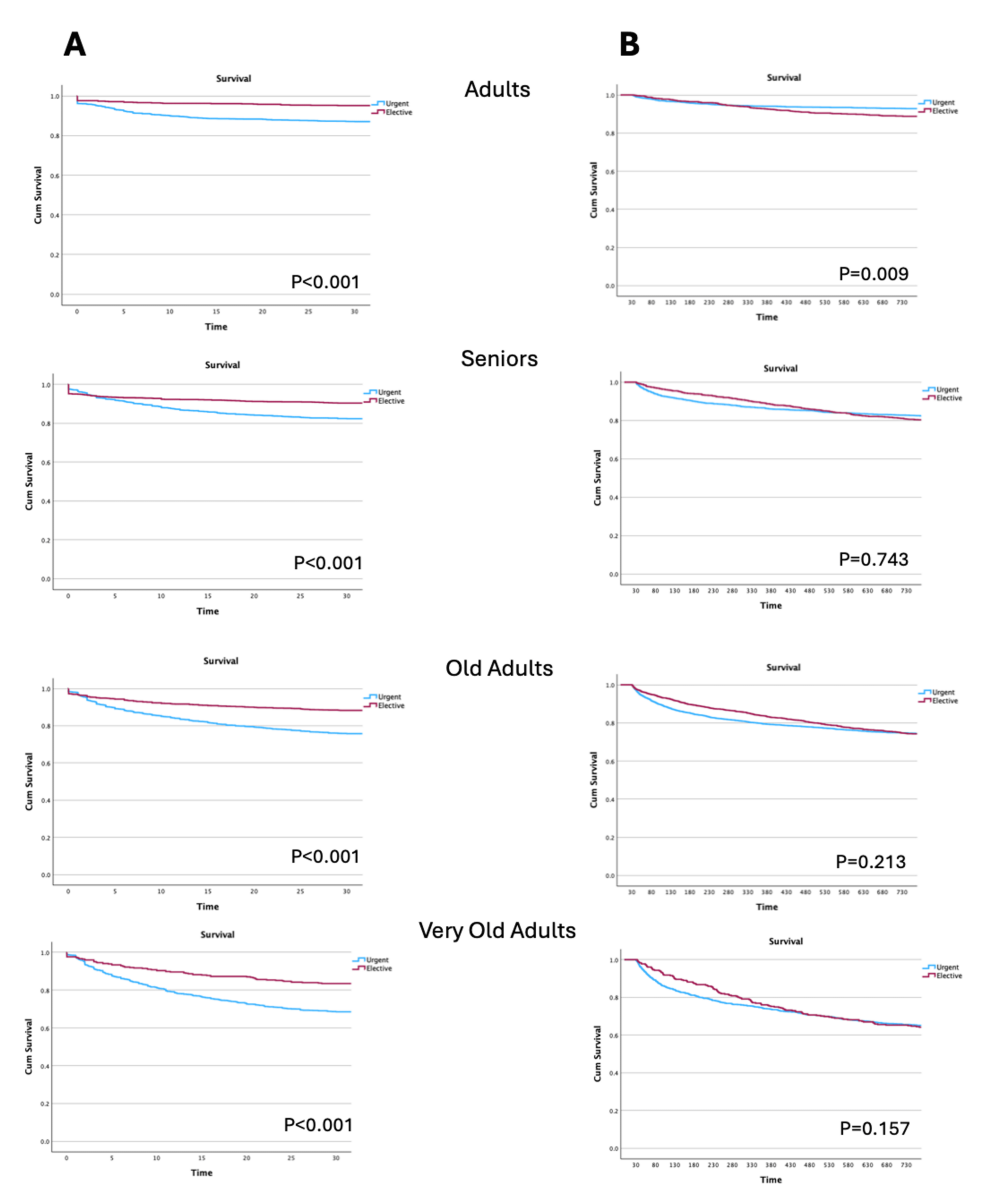
Survival curves according to the age group: survival in the first 30 days after admission (A) and from the 31st day until two years, addressing only patients who survived the first 30 days after surgery (B) Age group: adult: 18-50 years, senior: ≥50-65, old: ≥65-80, very old: ≥80

Short-term (first 30 days) and long-term (for patients who survived the first 30 days) survival, according to the age group, are presented in Figure 3. Patients submitted to an urgent surgery had higher short-term mortality, which was independent of the age group. However, this difference was no longer present in the long-term (from 30 days to two years) survival. Besides, in the younger cohort (less than 50 years old), a significantly higher long-term mortality was observed after elective surgery (Figure 3B).

Impact of surgery on the overall survival

We applied the Portuguese Instituto Nacional de Estatística tables of one-year death probability, which relate the gender and age of a population to the predicted number of expected deaths in a population. According to the gender and age of the patients in our cohort who underwent urgent surgery (before admission to the ICU) and were discharged alive from the hospital, the expected one-year mortality would be 2.6%. In the group of patients admitted after a planned, elective surgery, the one-year expected mortality would be 2.3% (according to their demographic characteristics). Admission to the ICU after a surgical procedure largely increased the probability of death (OR: 10.8, 95% CI: 9.0-12.9, after an urgent procedure; OR: 14.6, 95% CI: 11.3-18.8, after an elective surgery).

## Discussion

This study compared the trajectories of elective and urgent surgical patients admitted to the ICU during the postoperative period. This was a post hoc analysis of the CIMbA-LT study that included patients admitted for more than 24 hours to Portuguese ICUs between January 1, 2015, and June 30, 2019. All patients were followed for two years of follow-up and deemed dead or alive.

We unveiled a high short-term mortality of patients admitted after an urgent, non-scheduled surgery. This was consistent with the elevated admission severity, addressed by the SAPS II severity score. Conversely, elective surgical patients, while exhibiting lower initial mortality, displayed increased long-term mortality, particularly among younger cohorts.

The high risks of short-term mortality associated with unscheduled surgery have been previously described. Zhou et al. demonstrated that the 30-day survival of patients who underwent emergency surgery for colon or colon-rectal cancer was poorer than that of those who underwent elective surgery [16]. This was probably associated with delayed intervention, preoperative instability, and higher procedural risks [16-18]. Nevertheless, even very severe patients may have an acceptable long-term prognosis if they can overcome the acute insult [19].

Weissman and Klein highlight the critical differences between elective and emergency surgical patients in ICU settings, emphasizing their distinct clinical profiles and outcomes [20]. Elective surgery patients are generally healthier, undergo planned procedures, and experience lower complication and mortality rates. In contrast, emergency surgery patients often present with severe pre-existing conditions, require shorter but more urgent operations, need prolonged postoperative mechanical ventilation, and have extended ICU stays with higher mortality rates.

However, after clinical stabilization, recovery from the acute disease, and ICU discharge, the long-term prognosis may be improved. Nevertheless, a long duration of the operation (OR: 2.21, 95% CI: 1.27-3.83), a higher Anesthesiology Society of America (ASA) classification (OR: 2.37, 95% CI: 1.15-4.88), a higher Charlson Comorbidity Index (OR: 4.74, 95%CI: 3.15-7.14), and postoperative medical complications (OR: 1.61, 95% CI: 1.05-2.47) have all been associated with a worse outcome [21]. On the contrary, the long-term outcome of the elective patients is probably mostly related to the underlying chronic surgical disease, and a progressive decline in survival is expected. These patients may survive the immediate postoperative period but face long-term health challenges due to underlying surgical conditions or complications related to their surgical treatment. Nevertheless, long-term four-year survival, even for patients with complicated solid cancers, may be as high as 40% [22].

In our cohort, this difference in long-term prognosis was statistically significant in the younger population, possibly related to the best outcome of young patients who survived the first days after being submitted to a non-scheduled surgery. The main determinants of long-term outcomes of surgical patients seem to be age and comorbidities, and not the acute disease itself [23].

Differentiating these subpopulations is essential for optimizing resource allocation, tailoring postoperative care, and improving the interpretation of quality assessment data. This will ultimately lead to better patient management and more meaningful outcome comparisons. This is especially important, as the need for intensive care admission after a surgical procedure is associated with a very high risk of short- and long-term mortality when compared to the general population [21]. In our cohort, both urgent and elective surgical patients admitted to the ICU, despite being discharged alive from the hospital, have a roughly 10 times higher risk of dying within one year than a similar general population of the same age and gender. These findings were independent of the ICU despite significant differences in the admission criteria, either after elective or emergency surgery. Notably, the pattern was always similar: high early mortality of the urgent surgery patients and higher mortality of the elective surgery patients afterward. In the long-term follow-up, convergence was noted.

Our findings carry important implications for clinical practice. Firstly, they underscore the necessity of a stratified risk assessment upon ICU admission, considering individual patient factors such as age, underlying conditions, and sepsis. Secondly, patients submitted to elective or urgent surgeries have very distinct risk profiles and should be evaluated separately and receive tailored strategies after ICU and hospital discharge, to reduce long-term risks with structured outpatient follow-up programs.

Limitations and strengths

Our study has several limitations. First, the study relies on a retrospective, observational design, which may introduce selection bias and limit the ability to establish causal relationships. Second, our sample was restricted to a set of ICUs within a single country, and our population had a relatively high severity on admission, which may limit generalizability. Third, although the study accounts for severity scores (SAPS II) and the presence of sepsis, the classification of surgical patients was based on SAPS II criteria and may not be fully reproducible across different settings. Additionally, the analysis does not thoroughly examine other patient-specific factors, such as comorbidities, frailty, preoperative functional status, or the type and complexity of surgery, which may have influenced long-term outcomes and contributed to the observed mortality differences. Fourth, the study design did not allow evaluation of surgical complications or limitation-of-therapy decisions and therefore does not permit causal inference. Finally, this study does not provide detailed information on post-discharge care, rehabilitation, or outpatient follow-up, all of which could impact long-term outcomes. Variability in these factors could confound the observed differences in mortality between groups.

The study also has some strengths, namely, a large database covering most of the Portuguese territory, which allowed some robust conclusions.

We believe that our study offers important insights and encourages further research to build upon and validate these results.

## Conclusions

Elective and urgent surgical patients admitted to the ICU follow distinct short- and long-term outcome trajectories. Urgent surgical patients presented with greater severity at admission and experienced significantly higher short-term mortality, largely reflecting the acute physiological insult and the higher prevalence of sepsis. Conversely, among hospital survivors, those undergoing elective surgery exhibited an increased risk of long-term mortality, particularly the younger age groups, suggesting that factors beyond the immediate postoperative period influence survival.

Notably, admission to the ICU after elective and urgent surgery was associated with a markedly increased risk of mortality during the first year after discharge, compared to the general population. These findings emphasize that postoperative ICU patients should not be considered as a homogeneous group and reinforce the need for differentiated risk stratification and tailored post-discharge follow-up strategies to improve long-term outcomes.
